# PCSK9 Inhibition and Risk of Diabetes: Should We Worry?

**DOI:** 10.1007/s11883-022-01074-y

**Published:** 2022-11-16

**Authors:** Stefano Carugo, Cesare R. Sirtori, Alberto Corsini, Lale Tokgozoglu, Massimiliano Ruscica

**Affiliations:** 1 Department of Clinical Sciences and Community Health, Università Degli Studi Di Milano, Milan, Italy; 2 Fondazione Ospedale Maggiore IRCCS Policlinico Di Milano, Milan, Italy; 3 Department of Pharmacological and Biomolecular Sciences, Università Degli Studi Di Milano, Milan, Italy; 4 Hacettepe University, Ankara, Turkey

**Keywords:** PCSK9, New-onset diabetes, β-cells, Evolocumab, Alirocumab

## Abstract

**Purpose of Review:**

Since the clinical benefit of proprotein convertase subtilisin/kexin type 9 (PCSK9) inhibitors occurs in a setting of reducing low-density lipoprotein-cholesterol (LDL-C) to unprecedentedly low levels, it becomes of interest to investigate possible adverse effects pertaining to the risk of new-onset diabetes (NOD).

**Recent Findings:**

While safety results reported in either meta-analyses or cardiovascular outcome trials FOURIER (with evolocumab) and ODYSSEY (with alirocumab) did not rise the incidence of NOD, Mendelian randomization analyses were almost concordant in showing an increased risk of NOD. This evidence was in line with post-marketing safety reports highlighting that evolocumab and alirocumab were primarily related to mild hyperglycaemia rather than diabetes, with most of the hyperglycaemic events occurring during the first 6 months of treatment.

**Summary:**

Considering the different nature of genetic studies and of randomized controlled trials, with careful monitoring of patients, particularly in the earlier phases of treatment, and the identification of those more susceptible to develop NOD, treatment with PCSK9 inhibitors should be of minimal concern.

## Introduction

Atherosclerotic cardiovascular disease (ASCVD), contributing to more than 30% of the total global burden of disease, is still the leading cause of death and disability worldwide despite excellent pharmacological approaches and revascularizations [1]. Clinical and genetic studies unequivocally demonstrate that elevated low-density lipoprotein cholesterol (LDL-C) plays a causal role in the development and progression of ASCVD [2]. Thus, maintaining optimal lipid levels throughout life by keeping concentrations of LDL-C low to minimize the rate of progression of atherosclerotic plaques is a major strategy to reduce the risk of events [3]. The relationship between the extent of LDL-C lowering and ensuing cardiovascular (CV) risk reduction has been proven across different statin and non-statin therapies [4]. The relative risk reduction of major vascular events is proportional to LDL-C lowering for all drug classes, namely, statins, bile acid sequestrants, ezetimibe, proprotein convertase subtilisin/kexin type 9 (PCSK9) inhibitors and fibrates. The achieved lowering of LDL-C is directly linked to a reduced incidence of major ASCVD events [4]. However, although more than 30 years of clinical studies have shown that statin (in particular high-intensity statin) therapy reduces major vascular events in ASCVD patients, it is fundamental to bear in mind that combination therapies are advocated when LDL-C levels are not adequately controlled [5, 6].

A major therapeutic boost in the field of lipidology came from the approval of two fully human monoclonal antibodies (alirocumab (IgG1) and evolocumab (IgG2)) and, more recently, by a gene-silencing agent (inclisiran) against *PCSK9* [7, 8]. Alirocumab and evolocumab robustly decrease LDL-C by 50–60% on top of statins as well as the risk of major atherosclerotic vascular events [9, 10], with a continued effectiveness even with LDL-C below 40 mg/dL [11]. PCSK9 inhibitors and statins show similar effects on CV risk reduction per mmol/L reduction in LDL-C when the same duration of therapy is considered, namely, 14% (between 0 and 1 year of treatment), 17% (between 1 and 2 years of treatment) and 20% (between 2 and 3 years of treatment) [12]. This clinical trial evidence prompted a change in the LDL-C goals in Guidelines such as those from ESC/EAS, now recommending more stringent goals for high and very high-risk patients (e.g. < 55 mg/dL) [6] and those from ACC/AHA with an LDL-C target of < 70 mg/dL in patients with high-risk ASCVD [5].

Since these therapeutic instruments allow to reach unprecedented low levels of LDL-C, namely, < 30 mg/dL, it is of paramount importance to evaluate the long-term safety of very low LDL-C levels [13]. Indeed, the median follow-up of clinical trial participants in the two dedicated cardiovascular outcome trials testing evolocumab and alirocumab has been relatively short, respectively, 2.2 years [14] and 2.8 years [15]. Conversely, in statin trials, the median follow-up duration for cardiovascular outcome trials was between 4 and 5 years [16]. Since new-onset diabetes (NOD) with statins was evident many years after regulatory approval through meta-analyses of multiple trials [17], the present review will address the evidence pertaining to the risk of NOD associated with PCSK9 inhibition. To pursue this goal, preclinical, genetic and clinical evidence has been taken into consideration.

## Lesson Learnt from Statins

While there is a generally held view that the CV benefit of statins outweighs the risk of newly occurring NOD [18, 19], a hazard ratio (HR) of ≈1.1 has been found in the case of moderate dose and 1.2 for intensive statin therapy over a period of 5 years [19]. While a meta-analysis of 13 statin trials reported a 9% (95%CI: 1.02–1.17) increase in the odds ratio of developing NOD with a higher risk in older patients, it should be reminded that pre-diabetes represents a strong predictor of the development of NOD during a 5-year follow-up and that, compared to moderate-intensity statin therapy, the high-intensity statin approach raises the risk of NOD only in patients with pre-diabetes [20]. Genetic studies have provided significant help, in establishing the association between *3-Hydroxy-3-Methylglutaryl-CoA Reductase* (*HMGCR*) inhibition and odds of developing NOD. Indeed, individuals carrying an LDL-lowering allele in the *HMGCR* gene have a higher risk of NOD compared to non-carriers [21]. Mechanistically speaking, among possible hypotheses (reviewed elsewhere) [17, 22], the most accepted ones are: (a) raised insulin resistance, (b) reduction in insulin mediated glucose uptake in skeletal muscle and (c) antagonism on calcium channels in β-cells [23].

## Which Role Does PCSK9 Play in β-Cells?

In pancreatic β cells, the accumulation of cholesterol occurs mainly via LDL receptor (LDLR). Any genetic predisposition or pharmacological intervention raising the expression of LDLR is virtually associated with cholesterol overload in β cells, a process hypothetically dampening glucose-stimulated insulin secretion [24–26]. Excess cholesterol in β cells is then removed through the reverse cholesterol transport, a process mediated by different carriers, including the ATP-binding cassette transporter A1 [27].

Being mainly synthetized and released by the liver, PCSK9 represents one of the key regulators of LDL-C. Briefly, PCSK9 contains 3 distinct structural domains: the prodomain (aa 31–152), the catalytic subunit (aa 152–421) and the C-terminal Cys/His-rich domain (aa 453–692), each playing critical roles in the regulation of PCSK9 and of its intracellular traffic [28]. By means of the catalytic domain, PCSK9 interacts directly with the epidermal growth factor repeat A domain of the LDLR, a process favouring its degradation through an extracellular route implicating clathrin heavy chain–mediated endocytosis [29]. In recent years, however, molecular mechanisms beyond cholesterol lowering have been described. In particular Mendelian randomization analyses [30–32] and a phenome-wide association study [33] have highlighted a higher risk of developing NOD in carriers of genetic variants at the *PCSK9* locus that recapitulate the effects of therapeutic inhibition of PCSK9 on major blood lipid fractions and myocardial infarction.

### Preclinical Studies

If it is true that the use of murine models can help to dissect out some of the molecular mechanisms associated to a pathophysiological state, in the case of the link between PCSK9 and pancreatic β-cell function some contrasting results may be dependent upon the age and/or the genetic background of the models. While there is consensus on the identification of PCSK9 in the Langerhans’ islets [34], uncertainty remains relative to the exact location, some studies reporting protein expression of PCSK9 in δ-cells, with no detectable expression in α- and β-cells of *Pcsk9*^*−/−*^ mice [35], and others indicating that murine β-cells do express PCSK9 [34, 36]. In line with this latter, not only the expression of PCSK9 was found in human pancreatic β-cells [37, 38], but these cells were reported to secrete PCSK9.

Looking at the physiological role played by PCSK9, in fully *Pcsk9*^*−/−*^ mice, the expression of LDLR was raised in β cells [39], although it is important to note that PCSK9 regulates multiple cell surface receptors in pancreatic β-cells, namely, very low-density lipoprotein receptor levels, as well as levels of cluster of differentiation 36 (CD36), a fatty acid transporter [38].

Concerning glucose homeostasis, *Pcsk9*^*−/−*^ mice exhibit impaired glucose tolerance with an altered glucose-stimulated insulin secretion [40]. The defective glucose homeostasis subsequent to *Pcsk9* genetic deletion was due to an impaired insulin secretion rather than peripheral resistance; plasma insulin and C-peptide levels were significantly reduced, and the pancreatic insulin content was raised. This study highlighted the crucial role of LDLR in mediating such effects since the morphological and functional alterations of β-cells were normalized when analyses were repeated in double knockout mice for *Lldr* and *Pcsk9* [39]. Different conclusions were reached by Peyot et al. who generated a β-cell-specific KO Pcsk9 mice [36], noting that these animals displayed an increased basal and stimulated insulin secretion compared to controls. However, the deletion of PCSK9 in endocrine pancreas precursors and mature β- and δ-cells in *Pdx1Cre*^+^ mice led to an impairment in insulin secretion, an effect mediated by the reorganization of the secretory machinery of β-cells via the involvement of LDLR-cholesterol axis [34].

## Does PCSK9 Raise the Risk of NOD? Genetic and Clinical Evidence

### Mendelian Randomization Analyses and Genetic Tools

Out of four studies evaluating the impact of inherited variants in the genes encoding PCSK9 that recapitulate the effect of PCSK9 pharmacological inhibition, three found a rise in the risk of NOD [30–32] and one a neutral effect [41]. Variants in *PCSK9* had approximately the same effect as variants in 3-hydroxy-3-methylglutaryl-coenzyme A reductase (*HMGCR*) on the risk of diabetes per unit decrease in the LDL-C [30]. For each 10 mg/dL drop in LDL-C, OR was 1.11 (95% CI, 1.04–1.19) for *PCSK9* and 1.13 (95% CI, 1.06–1.20) for *HMGCR* [30]. PCSK9 variants associated with lower LDL-C were also associated with higher fasting glucose concentrations and an increased risk of NOD. Each 38.7 mg/dL lower LDL-C corresponded to 0.09 mmol/L (1.62 mg/dL) higher fasting glucose (95%CI 0.02–0.15) and a 29% rise in the odds of NOD (OR = 1.29, 95%CI 1.11–1.50) [31]. A more recent phenome-wide association study resulting in a sample size of 320,170 individuals indicated an increased risk of NOD (OR = 1.29; 95%CI 1.11–1.50) [33]. Overall, these conclusions were confirmed in a meta-analysis of genetic association studies including 50,775 individuals with type 2 diabetes and 270,269 controls. LDL-C lowering alleles at *PCSK9* were associated with a higher risk of diabetes, namely, each 1 mmol/L (38.7 mg/dL) genetically predicted reduction in LDL-C led to an odds ratio of 1.19 (95% CI, 1.02–1.38) [32]. Similar conclusions were reached in a cohort of 75,441 individuals of Asian descent. Analysis of seven *PCSK9* variants showed an inverse association between genetically determined LDL-C levels and the prevalence of NOD. A reduction of 38.7 mg/dL of LDL-C was associated with an increased odds ratio of 1.61 (95%CI 1.04–1.29) [42]. Conversely, a 2 × 2 factorial Mendelian randomization study including 425,354 participants from the UK Biobank did not observe an association between lower PCSK9 concentrations and type 2 diabetes [41]. Similar conclusions were reached by a Mendelian randomization analysis evaluating the causal effect of various lipid traits on type 2 diabetes liability in roughly 70,000 individuals of African ancestry (odds ratio = 0.94; 95%CI 0.82–1.08) [43].

### Clinical Evidence

Since the clinical benefit of PCSK9 inhibitors seen in the FOURIER (Further cardiovascular OUtcomes Research with PCSK9 Inhibition in subjects with Elevated Risk) and ODYSSEY (ODYSSEY Outcomes: Evaluation of Cardiovascular Outcomes After an Acute Coronary Syndrome During Treatment With Alirocumab) outcome trials occurred in a setting of reducing LDL-C to unprecedentedly low levels (e.g. < 15 mg/dL), it has become of interest to investigate offsetting adverse effects (Table 1) [44].

**Table 1:** Risk of new-onset diabetes

Mendelian randomization analyses	
Ference [30]	OR 1.11 (95% CI, 1.04 to 1.19) for each 10 mg/dL decrease in LDL-C in carriers of *PCSK9* loss-of-function variants
Schmidt [31]	Combined analyses of four independent *PCSK9* variants (rs11583680, rs11591147, rs2479409 and rs11206510) scaled to 1 mmol/L lower LDL-C showed an OR for type 2 diabetes of 1·29 (1.11 to 1.50)
Lotta [32]	LDL-C–lowering alleles in or near *PCSK9* the OR for type 2 diabetes per 1-mmol/L genetically predicted reduction in LDL-C was 1.19 (95%CI, 1.02–1.38)
Cupido [41]	OR 1.00 (95%CI 0.97–1.02)
Cardiovascular outcome trials	
Sabatine [46]	HR 1.05 (95%CI 0.94–1.17) in patients without diabetes at baseline (FOURIER). 8% (663 of 8256) in the evolocumab group developed diabetes after randomization vs 7.6% (631 of 8254) in the placebo group
O’Donoghue [47••]	FOURIER-OLE evaluated the long-term safety of evolocumab (for over > 8 years). Placebo phase FOURIER (2.3%); Evolocumab phase FOURIER (1.8%) and Evolocumab phase FOURIER and OLE (1.2%)
Ray [52]	HR 1.00 (95%CI 0.89–1.11) among patients without diabetes at baseline (ODYSSEY OUTCOMES). Overall, 648 patients (9.6%) in the alirocumab group developed diabetes after randomization vs 676 patients (10.1%) in the placebo group
FDA adverse event reporting system	
Goldman [65]	Treatment with PCSK9 inhibitors was associated with increased reporting of hyperglycaemia; adjusted ROR = 1.14 (95%CI 1.07–1.22)
Ji [66]	PCSK9 inhibitors raise blood glucose (ROR = 1.86 (95%CI 1.68–2.05))

1 mmol/L of LDL-C corresponds to 38.7 mg/dL

*FOURIER* Further cardiovascular OUtcomes Research with PCSK9 Inhibition in subjects with Elevated Risk, *FOURIER-OLE* FOURIER open-label extension program, *ODYSSEY OUTCOMES* Evaluation of Cardiovascular Outcomes After an Acute Coronary Syndrome During Treatment With Alirocumab, *LDL-C* low-density lipoprotein cholesterol, *HR* hazard ratio, *OR* odds ratio, *PCSK9* proprotein convertase subtilisin/kexin type 9, *ROR* reporting odds ratio

### Evolocumab

**Fig. 1:**
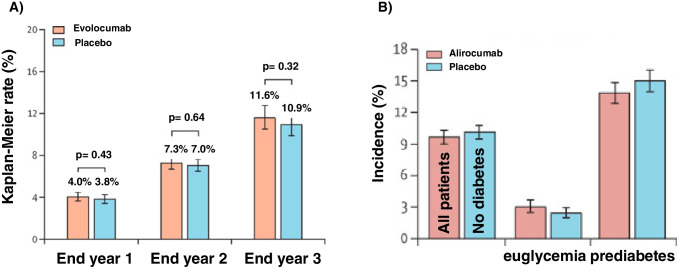
(**A)** Cumulative incidence of new-onset diabetes at the end of 1, 2 and 3 years of follow-up in the FOURIER study, among patients without diabetes at baseline. Error bars are 95% Cis. (Fig. 1A Reproduced from: Sabatine MS et al. Lancet Diabetes Endocrinol 2017, 5(12):941–950, with permission from Elsevier) [46]. **B)** Post-randomization new-onset diabetes in ODYSSEY OUTCOME trial. Error bars are 95% CIs. (Fig. 1B Reproduced from: Ray KK et al. Lancet Diabetes Endocrinol 2019, 7(8):618–628, with permission from Elsevier) [52]

In a secondary prespecified analysis of the FOURIER (Further cardiovascular OUtcomes Research with PCSK9 Inhibition in subjects with Elevated Risk) study, among 25,982 patients of the trial, the impact of progressively lower LDL-C concentrations achieved, and clinical efficacy and safety were assessed. While a monotonic relationship was found between achieved LDL-C and major cardiovascular outcomes, pertaining safety no differences were noted in NOD [45]. A confirmation came also by the results of a further post hoc analysis aimed at assessing the efficacy and safety of evolocumab in patients with and without diabetes [46]. Overall, the trial showed that there was neither an increment in the cumulative incidence of NOD in the evolocumab and placebo treatment groups at the end of 1, 2 and 3 years of follow-up (Fig. 1A) nor changes in fasting plasma glucose and HbA_1c_ overtime. Specifically, evolocumab did not increase the risk of NOD in patients without diabetes at baseline (hazard ratio (HR) = 1.05, 95%CI 1.05 (0.94–1.17)), including those with prediabetes (HR = 1.00, 95%CI 0.89–1.13). Of note, the safety of a longer exposure to evolocumab (median follow-up was 7.1 years) was the aim of the FOURIER open-label extension program (FOURIER-OLE). The trial enrolled 6635 patients of whom 3355 (50.6%) originally randomized in the parent trial to evolocumab and 3280 to placebo. Maximum exposure to evolocumab during parent plus FOURIER-OLE was 8.4 years. The overall annualized incidence rate of NOD was not higher among patients in the OLE (1.2%) compared to those in the placebo arm of the original trial (2.3%) [47••]. The absence of NOD risk was also found in the BANTING (EvolocumaB efficAcy aNd safeTy IN type 2 diabetes mellitus on backGround statin therapy) study showing that among participants with type 2 diabetes there were no notable differences in fasting glucose or HbA_1c_ levels between those randomized to evolocumab or to placebo [48]. A further reassurance on glucose homeostasis came from pooled 1-year (48-week) data evaluating participants who had completed an evolocumab parent study before entering an open-label extension trial [49]. Among these, the HAUSER-OLE (Safety, Tolerability and Efficacy of Evolocumab (AMG 145) in Children With Inherited Elevated Low-density Lipoprotein Cholesterol (Familial Hypercholesterolemia)) study showed that paediatric patients with heterozygous familial hypercholesterolaemia did not develop diabetes during a follow-up of 80 weeks [50••].

### Alirocumab

A pooled analysis of 14 trials (both phase 2 and phase 3 studies) in which LDL-C levels reached < 25 or < 15 mg/dL was run to test the association between LDL-C with the increase in overall treatment emergent rate of adverse events. Propensity analysis of the risk of NOD in alirocumab-treated patients with two or more consecutive LDL-C values < 25 mg/dL showed no risk of diabetes or diabetic complications event (regardless of baseline status), namely, HR = 1.09 (95%CI 0.72–1.65) for LDL-C < 25 vs ≥ 25 mg/dL [51]. Glycaemic safety of alirocumab was confirmed in a secondary analysis of the ODYSSEY OUTCOME trial showing that the risk of developing NOD in patients without diabetes at baseline did not differ between alirocumab and placebo (Fig. 1B). The HR for NOD for patients with normoglycemia or prediabetes was 0.95 (95%CI 0.85–1.05) [52].

A further post hoc analysis evaluating the efficacy of alirocumab according to metabolic risk factors concluded that the incidence of NOD was similar in the alirocumab and placebo groups [53]. The administration of alirocumab did not show clinically meaningful effect on HbA_1c_, or change in number of glucose-lowering agents in ODYSSEY DM-DYSLIPIDEMIA trial enrolling individuals with type 2 diabetes and mixed dyslipidaemia not optimally managed by maximally tolerated statins [54].

### Meta-Analyses of Clinical Studies

A meta-analysis of 20 clinical trials (phase 2 and phase 3 studies) including 68,123 participants within median follow-up of 78 weeks showed a significantly increased fasting blood glucose (1.88 mg/dL; 0.1 mmol/L) and HbA_1c_ (0.032%) in patients given PCSK9 inhibitors. In particular, the imbalance in glucose homeostasis rose stepwise according to the duration of treatment starting to be evident at mean follow-up of 1.5 years. Despite this, the incidence of diabetes appeared not to be raised (relative risk = 1.04; 95%CI 0.96–1.13) as was for the worsening of diabetes [55]. However, these findings were quite disputable since in the analyses the authors included SPIRE trial with bocucizumab, a monoclonal antibody which was withdrawn due to the development of antidrug antibodies [56]. Indeed, it seems that when the analysis is performed without SPIRE trials, PCSK9 inhibitors have no effect on circulating fasting plasma glucose FBG or HbA_1c_ levels with no impact of treatment duration or percent change of LDL-C cholesterol [57].

**Fig. 2:**
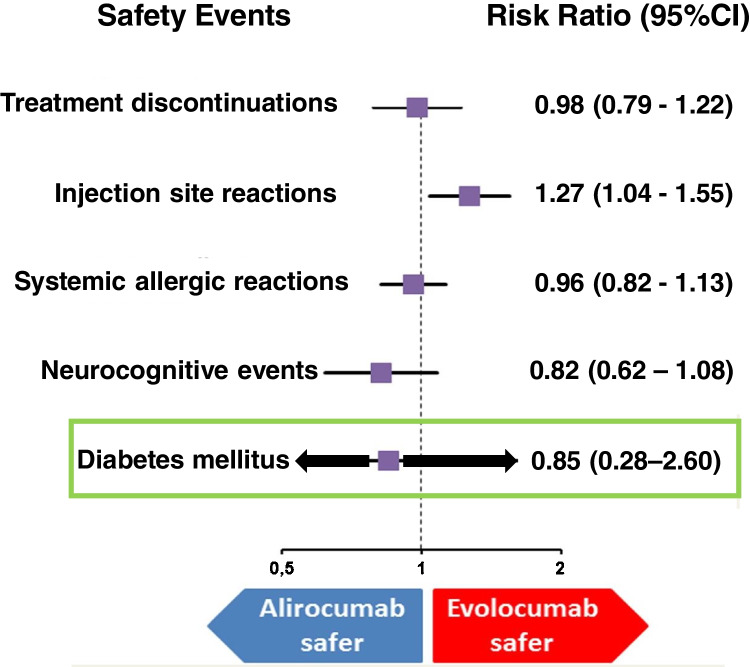
Indirect comparisons of the safety of evolocumab and alirocumab. (Reproduced from: Guedeney P et al.: Eur Heart J Cardiovasc Pharmacother 2021, 7(3):225–235, by permission of Oxford University Press) [64]

Reassuring the growing number of patients that need to take potent lipid-lowering medications, there are data of a meta-analysis of RCTs evaluating statins and statins/PCSK9 inhibitors as intervention in 163,688 nondiabetic patients randomly assigned to more intensive (*n* = 83,123) or less intensive (*n* = 80,565) lipid lowering therapy. Neither LDL-C lowering (38.7 mg/dL) nor the use of PCSK9 inhibitors was associated with the incidence of diabetes, respectively, risk ratio = 1.07 (95%CI 1.03–1.11) and risk ratio = 1.00 (95%CI 0.93–1.07) [58]. In line with this evidence, other meta-analyses failed to detect a raised risk of hyperglycaemia or diabetes [59–61]. To quantify the safety of PCSK9 inhibitors with a specific focus on type 2 diabetes was also the topic of a Cochrane analysis. The following comparisons were assessed: alirocumab vs placebo (odds ratio = 0.96, 95%CI 0.86–1.07), evolocumab vs placebo (odds ratio = 1.05, 95%CI 0.94–1.17), alirocumab vs other lipid lowering treatments (odds ratio = 0.28, 95%CI 0.05–1.55) and evolocumab vs alternative lipid lowering treatments (odds ratio = 3.52, 95%CI 0.18–68.33) [62]. In line with this topic, later studies, comparing treatment with alirocumab or evolocumab vs placebo or other lipid lowering therapies, did not appear to determine a clear-cut rise of NOD. This risk was 1.92 vs 1.93 per 100 patients-years, risk ratio = 1.00 (95%CI 0.93–1.07; *p* = 0.97) [63]. Similar conclusions were reached when the analysis was repeated comparing alirocumab or evolocumab to placebo with consistent and maximally tolerated background lipid-lowering therapy. No differences were observed in the risk of diabetes or worsening of pre-existing diabetes (relative risk = 0.85; 95%CI 0.28–2.60) (Fig. 2) [64].

### Post-Marketing Safety Reports

Real-world experience with PCSK9 inhibitors is still in its infancy, with neurocognitive adverse events and diabetes remaining debated long-term safety issues. The most significant large-scale evaluation on the association of hyperglycaemic disorders with PCSK9 inhibitors has been carried out by accessing the Food and Drug Administration Adverse Event Reporting System (FAERS), a database providing reliable data for the early detection of rare adverse events and continuous monitoring of recently marketed medications, especially monoclonal antibodies. Among the 7,295,624 patients retained in the analysis (after appropriate filtering, e.g. unclear adverse effect reporting), 87,724 reports were identified from PCSK9 inhibitors recipients and similar numbers from statin or ezetimibe-treated patients [65]. There was a raised reporting of hyperglycaemias associated with PCSK9 inhibitors treatment compared to the full database (*n* = 1841/87,274 (2.1%); adjusted-reporting odds ratio (ROR) = 1.14 (1.07–1.22). However, when a head-to-head comparison was conducted between evolocumab and alirocumab, a disproportionate reporting of hyperglycaemia was found with evolocumab (*n* = 1587/71748 (2.1%), adjusted ROR 1.24 (1.15–1.32), not with alirocumab (*n* = 254/15,976; adjusted ROR = 0.73 (0.60–0.88)) [65]. However, PCSK9 inhibitors were primarily related to mild hyperglycaemias (*n* = 1587/87,724 (1.67%), adjusted ROR 1.48 (1.36–1.62)) rather than to diabetes (*n* = 372/87,724 (0.42%), adjusted ROR 0.67 (0.67–0.074)) [65]. A further analysis on the same database, but extended to March 2021, reported a blood glucose increase associated to PCSK9 inhibitors: a ROR of 1.86 (1.68–2.05) was found when evolocumab and alirocumab were considered as a whole; a ROR of 1.55 (1.29–1.85) was found in the case of alirocumab and a ROR of 2.03 (1.80–2.29) in the case of evolocumab [66]. Interestingly, diabetic patients given PCSK9 inhibitors experienced hyperglycaemia more frequently than non-diabetic patients, namely, 11.3% vs 2.1% in the case of evolocumab and 9.9% vs 1.3% in the case of alirocumab.

**Fig. 3:**
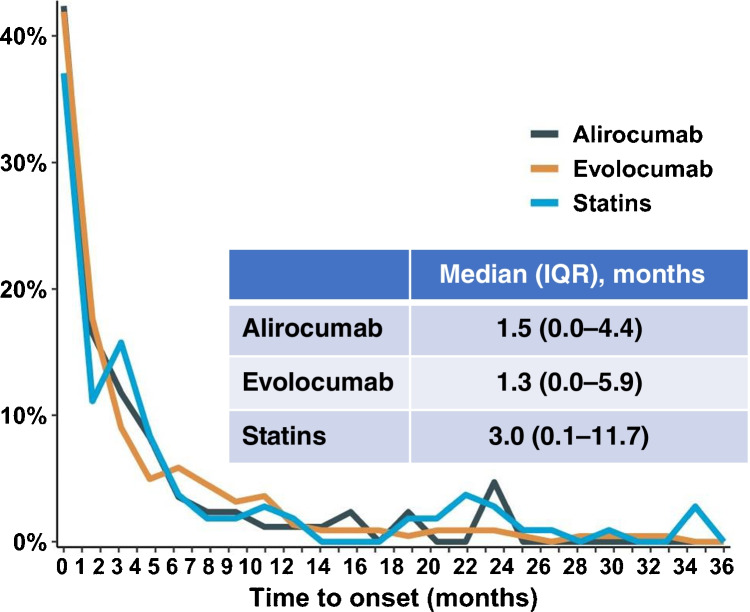
Clinical characteristics of hyperglycaemic adverse events with PCSK9 inhibitor and statin treatments. Months from treatment initiation to hyperglycaemic adverse event onset. IQR, interquartile range. (Reproduced from: Goldman A et al. Eur J Prev Cardiol 2022, 29(9):1334–1342, by permission of Oxford University Press) [65]

Besides the evaluation of the relationship between PCSK9 inhibitors and the risk of NOD, it is of interest to understand the lag frame required to diabetes to manifest. Most of the hyperglycaemic events occurred during the first 6 months of treatment, with a median time to onset of 1 month vs 3 months when statins were considered (Fig. 3) [65]. It seems that this difference is related to the known time of LDL-C reduction which is between 14 and 21 days with PCSK9 inhibitors and 4–6 weeks with statins [67]. Finally, improvement or resolution after drug withdrawal was found, whereas the rechallenge led to a reoccurrence of the adverse event [65].

## Conclusions

In the complex metabolic control of patients with ASCVD presenting with lipid/lipoprotein abnormalities, the possibly increased incidence of diabetes frequently raises concern among cardiologists and attending general practitioners. Although the genetics was concordant in showing an increased risk of NOD, this evidence needs to be interpreted in the strict context of this tool. Indeed, loss-of-function variants in *PCSK9* are used as proxies to understand safety and efficacy of living with very low levels of LDL-C in the long term, an exchangeability that is not guaranteed in clinical trials [68]. Despite sometimes being referred to as “nature’s randomized trial,” a Mendelian randomization study cannot be used to replace a randomized trial but instead provides complementary information [69]. Moreover, data extrapolated from Biobanks, e.g. the UK Biobank, refer to a healthy population with a low prevalence of glycaemic abnormality at baseline, whereas in clinical trials the effects of pharmacological PCSK9 inhibition on incident diabetes are estimated in patients that could already suffer from hyperglycaemia or diabetes. These inherited differences could partially explain the reason why clinical trials have not shown any effect of PCSK9 inhibitors on increasing the fasting plasma glucose or NOD whereas real-world experience found an association between the use of PCSK9 inhibitors and hyperglycaemia, although primarily related to mild hyperglycaemia rather than diabetes. In line with this hypothesis are the data of a prospective analysis of 2 cohorts one from France (100% with pre-diabetes; follow-up 4 years) and another from Brazil (27% with pre-diabetes; follow-up 5 years) that reported no association between PCSK9 plasma concentrations with NOD [70]. Overall, the careful monitoring of patients, particularly in the earlier phases of treatment, as well as the identification of individuals more susceptible to develop NOD, should render PCSK9 inhibitors of minimal worry to both physicians and patients.

## References

[1] Cardiovascular diseases https://www.who.int/health-topics/cardiovascular-diseases#tab=tab_1. Accessed 7 Nov 2022

[2] Ference BA, Ginsberg HN, Graham I (2017). Low-density lipoproteins cause atherosclerotic cardiovascular disease. 1. Evidence from genetic, epidemiologic, and clinical studies. A consensus statement from the European Atherosclerosis Society Consensus Panel. Eur Heart J.

[3] Ference BA, Graham I, Tokgozoglu L, Catapano AL (2018). Impact of lipids on cardiovascular health: JACC health promotion series. J Am Coll Cardiol.

[4] Silverman MG, Ference BA, Im K (2016). Association between lowering LDL-C and cardiovascular risk reduction among different therapeutic interventions: a systematic review and meta-analysis. JAMA.

[5] Grundy SM, Stone NJ, Bailey AL (2019). 2018 AHA/ACC/AACVPR/AAPA/ABC/ACPM/ADA/AGS/APhA/ASPC/NLA/PCNA guideline on the management of blood cholesterol: a report of the American College of Cardiology/American Heart Association Task Force on Clinical Practice Guidelines. Circulation.

[6] Mach F, Baigent C, Catapano AL (2020). 2019 ESC/EAS Guidelines for the management of dyslipidaemias: lipid modification to reduce cardiovascular risk. Eur Heart J.

[7] Ruscica M, Ferri N, Santos RD, Sirtori CR, Corsini A (2021). Lipid lowering drugs: present status and future developments. Curr Atheroscler Rep.

[8] Pirillo A, Catapano AL (2022). Inclisiran: how widely and when should we use it?. Curr Atheroscler Rep.

[9] Preiss D, Tobert JA, Hovingh GK, Reith C (2020). Lipid-modifying agents, from statins to PCSK9 inhibitors: JACC Focus Seminar. J Am Coll Cardiol.

[10] Gouni-Berthold I, Schwarz J, Berthold HK (2022). PCSK9 monoclonal antibodies: new developments and their relevance in a nucleic acid-based therapy era. Curr Atheroscler Rep.

[11] Marston NA, Giugliano RP, Park JG (2021). Cardiovascular benefit of lowering low-density lipoprotein cholesterol below 40 mg/dL. Circulation.

[12] Ference BA, Cannon CP, Landmesser U, Luscher TF, Catapano AL, Ray KK (2018). Reduction of low density lipoprotein-cholesterol and cardiovascular events with proprotein convertase subtilisin-kexin type 9 (PCSK9) inhibitors and statins: an analysis of FOURIER, SPIRE, and the Cholesterol Treatment Trialists Collaboration. Eur Heart J.

[13] Karagiannis AD, Mehta A, Dhindsa DS (2021). How low is safe? The frontier of very low (<30 mg/dL) LDL cholesterol. Eur Heart J.

[14] Sabatine MS, Giugliano RP, Keech AC (2017). Evolocumab and clinical outcomes in patients with cardiovascular disease. N Engl J Med.

[15] Schwartz GG, Steg PG, Szarek M (2018). Alirocumab and cardiovascular outcomes after acute coronary syndrome. N Engl J Med.

[16] Cholesterol Treatment Trialists C, Baigent C, Blackwell L (2010). Efficacy and safety of more intensive lowering of LDL cholesterol: a meta-analysis of data from 170,000 participants in 26 randomised trials. Lancet.

[17] Ruscica M, Ferri N, Banach M, Sirtori CR, Corsini A. Side effects of statins-from pathophysiology and epidemiology to diagnostic and therapeutic implications. Cardiovasc Res. 2022;3:cvac020. 10.1093/cvr/cvac020. 10.1093/cvr/cvac020 35238338

[18] Mach F, Ray KK, Wiklund O (2018). Adverse effects of statin therapy: perception vs. the evidence—focus on glucose homeostasis, cognitive, renal and hepatic function, haemorrhagic stroke and cataract. Eur Heart J.

[19] Newman CB, Preiss D, Tobert JA (2019). Statin Safety and associated adverse events: a scientific statement from the American Heart Association. Arterioscler Thromb Vasc Biol.

[20] Kohli P, Waters DD, Nemr R (2015). Risk of new-onset diabetes and cardiovascular risk reduction from high-dose statin therapy in pre-diabetics and non-pre-diabetics: an analysis from TNT and IDEAL. J Am Coll Cardiol.

[21] Swerdlow DI, Preiss D, Kuchenbaecker KB (2015). HMG-coenzyme A reductase inhibition, type 2 diabetes, and bodyweight: evidence from genetic analysis and randomised trials. Lancet.

[22] Ruscica M, Macchi C, Morlotti B, Sirtori CR, Magni P (2014). Statin therapy and related risk of new-onset type 2 diabetes mellitus. Eur J Intern Med.

[23] Yada T, Nakata M, Shiraishi T, Kakei M (1999). Inhibition by simvastatin, but not pravastatin, of glucose-induced cytosolic Ca2+ signalling and insulin secretion due to blockade of L-type Ca2+ channels in rat islet beta-cells. Br J Pharmacol.

[24] Roehrich ME, Mooser V, Lenain V (2003). Insulin-secreting beta-cell dysfunction induced by human lipoproteins. J Biol Chem.

[25] Brunham LR, Kruit JK, Pape TD (2007). Beta-cell ABCA1 influences insulin secretion, glucose homeostasis and response to thiazolidinedione treatment. Nat Med.

[26] Rutti S, Ehses JA, Sibler RA (2009). Low- and high-density lipoproteins modulate function, apoptosis, and proliferation of primary human and murine pancreatic beta-cells. Endocrinology.

[27] Perego C, Da Dalt L, Pirillo A, Galli A, Catapano AL, Norata GD (2019). Cholesterol metabolism, pancreatic beta-cell function and diabetes. Biochim Biophys Acta Mol Basis Dis.

[28] Seidah NG, Prat A (2022). The Multifaceted Biology of PCSK9. Endocr Rev.

[29] Macchi C, Ferri N, Sirtori CR, Corsini A, Banach M, Ruscica M (2021). Proprotein convertase subtilisin/kexin type 9: a view beyond the canonical cholesterol-lowering impact. Am J Pathol.

[30] Ference BA, Robinson JG, Brook RD (2016). Variation in PCSK9 and HMGCR and risk of cardiovascular disease and diabetes. N Engl J Med.

[31] Schmidt AF, Swerdlow DI, Holmes MV (2017). PCSK9 genetic variants and risk of type 2 diabetes: a mendelian randomisation study. Lancet Diabetes Endocrinol.

[32] Lotta LA, Sharp SJ, Burgess S (2016). Association between low-density lipoprotein cholesterol-lowering genetic variants and risk of type 2 diabetes: a meta-analysis. JAMA.

[33] Schmidt AF, Holmes MV, Preiss D (2019). Phenome-wide association analysis of LDL-cholesterol lowering genetic variants in PCSK9. BMC Cardiovasc Disord.

[34] Marku A, Da Dalt L, Galli A*, et al*: Pancreatic PCSK9 controls the organization of the beta-cell secretory pathway via LDLR-cholesterol axis. Metabolism 2022:155291. 10.1016/j.metabol.2022.155291 35981632

[35] Langhi C, Le May C, Gmyr V (2009). PCSK9 is expressed in pancreatic delta-cells and does not alter insulin secretion. Biochem Biophys Res Commun.

[36] Peyot ML, Roubtsova A, Lussier R (2021). Substantial PCSK9 inactivation in beta-cells does not modify glucose homeostasis or insulin secretion in mice. Biochim Biophys Acta Mol Cell Biol Lipids.

[37] Ramin-Mangata S, Thedrez A, Nativel B (2021). Effects of proprotein convertase subtilisin kexin type 9 modulation in human pancreatic beta cells function. Atherosclerosis.

[38] Saitoski K, Ryaboshapkina M, Hamza GM (2022). Proprotein convertase PCSK9 affects expression of key surface proteins in human pancreatic beta cells via intracellular and extracellular regulatory circuits. J Biol Chem.

[39] Da Dalt L, Ruscica M, Bonacina F (2019). PCSK9 deficiency reduces insulin secretion and promotes glucose intolerance: the role of the low-density lipoprotein receptor. Eur Heart J.

[40] Mbikay M, Sirois F, Mayne J (2010). PCSK9-deficient mice exhibit impaired glucose tolerance and pancreatic islet abnormalities. FEBS Lett.

[41] Cupido AJ, Reeskamp LF, Hingorani AD (2022). Joint genetic inhibition of PCSK9 and CETP and the association with coronary artery disease: a factorial Mendelian randomization study. JAMA Cardiol.

[42] Hsu LA, Teng MS, Wu S, Chou HH, Ko YL: Common and rare PCSK9 variants associated with low-density lipoprotein cholesterol levels and the risk of diabetes mellitus: a Mendelian randomization study. Int J Mol Sci 2022, 23(18). 10.3390/ijms231810418 PMC9499600 36142332

[43] Soremekun O, Karhunen V, He Y (2022). Lipid traits and type 2 diabetes risk in African ancestry individuals: a Mendelian randomization study. EBioMedicine.

[44] Sabatine MS (2019). PCSK9 inhibitors: clinical evidence and implementation. Nat Rev Cardiol.

[45] Giugliano RP, Pedersen TR, Park JG (2017). Clinical efficacy and safety of achieving very low LDL-cholesterol concentrations with the PCSK9 inhibitor evolocumab: a prespecified secondary analysis of the FOURIER trial. Lancet.

[46] Sabatine MS, Leiter LA, Wiviott SD (2017). Cardiovascular safety and efficacy of the PCSK9 inhibitor evolocumab in patients with and without diabetes and the effect of evolocumab on glycaemia and risk of new-onset diabetes: a prespecified analysis of the FOURIER randomised controlled trial. Lancet Diabetes Endocrinol.

[47] •• O'Donoghue ML, Giugliano RP, Wiviott SD*,* et al: Long-term evolocumab in patients with established atherosclerotic cardiovascular disease. Circulation. 2022;146(15):1109–19. **This study addressed the salient issue regarding the durability of PCSK9 inhibitor-associated benefit and safety. There was no observed trend toward an increase in the incidence of any of the adverse events of interest over time (> 8 years).** 10.1161/CIRCULATIONAHA.122.061620 36031810

[48] Rosenson RS, Daviglus ML, Handelsman Y (2019). Efficacy and safety of evolocumab in individuals with type 2 diabetes mellitus: primary results of the randomised controlled BANTING study. Diabetologia.

[49] Sattar N, Toth PP, Blom DJ (2017). Effect of the proprotein convertase subtilisin/kexin type 9 inhibitor evolocumab on glycemia, body weight, and new-onset diabetes mellitus. Am J Cardiol.

[50] Santos RD, Ruzza A, Hovingh GK (2022). Paediatric patients with heterozygous familial hypercholesterolaemia treated with evolocumab for 80 weeks (HAUSER-OLE): a single-arm, multicentre, open-label extension of HAUSER-RCT. Lancet Diabetes Endocrinol.

[51] Robinson JG, Rosenson RS, Farnier M (2017). Safety of very low low-density lipoprotein cholesterol levels with alirocumab: pooled data from randomized trials. J Am Coll Cardiol.

[52] Ray KK, Colhoun HM, Szarek M (2019). Effects of alirocumab on cardiovascular and metabolic outcomes after acute coronary syndrome in patients with or without diabetes: a prespecified analysis of the ODYSSEY OUTCOMES randomised controlled trial. Lancet Diabetes Endocrinol.

[53] Ostadal P, Steg PG, Poulouin Y (2022). Metabolic risk factors and effect of alirocumab on cardiovascular events after acute coronary syndrome: a post-hoc analysis of the ODYSSEY OUTCOMES randomised controlled trial. Lancet Diabetes Endocrinol.

[54] Ray KK, Leiter LA, Muller-Wieland D (2018). Alirocumab vs usual lipid-lowering care as add-on to statin therapy in individuals with type 2 diabetes and mixed dyslipidaemia: the ODYSSEY DM-DYSLIPIDEMIA randomized trial. Diabetes Obes Metab.

[55] de Carvalho LSF, Campos AM, Sposito AC (2018). Proprotein convertase subtilisin/kexin type 9 (PCSK9) inhibitors and incident type 2 diabetes: a systematic review and meta-analysis with over 96,000 patient-years. Diabetes Care.

[56] Ferri N, Corsini A, Sirtori CR, Ruscica M (2017). Bococizumab for the treatment of hypercholesterolaemia. Expert Opin Biol Ther.

[57] Cao YX, Li JJ: Comment on de Carvalho et al. Proprotein convertase subtilisin/kexin type 9 (PCSK9) inhibitors and incident type 2 diabetes: a systematic review and meta-analysis with over 96,000 patient-years. Diabetes Care 2018;41:364–367. Diabetes Care 2018, 41(4):e69. 10.2337/dc17-2563 29559466

[58] Khan SU, Rahman H, Okunrintemi V (2019). Association of lowering low-density lipoprotein cholesterol with contemporary lipid-lowering therapies and risk of diabetes mellitus: a systematic review and meta-analysis. J Am Heart Assoc.

[59] Monami M, Sesti G, Mannucci E (2019). PCSK9 inhibitor therapy: a systematic review and meta-analysis of metabolic and cardiovascular outcomes in patients with diabetes. Diabetes Obes Metab.

[60] Chiu SW, Pratt CM, Feinn R, Chatterjee S (2020). Proprotein convertase subtilisin/kexin type 9 inhibitors and ezetimibe on risk of new-onset diabetes: a systematic review and meta-analysis of large, double-blinded randomized controlled trials. J Cardiovasc Pharmacol Ther.

[61] Li J, Du H, Wang Y (2022). Safety of proprotein convertase subtilisin/kexin 9 inhibitors: a systematic review and meta-analysis. Heart.

[62] Schmidt AF, Carter JL, Pearce LS (2020). PCSK9 monoclonal antibodies for the primary and secondary prevention of cardiovascular disease. Cochrane Database Syst Rev.

[63] Guedeney P, Giustino G, Sorrentino S (2022). Efficacy and safety of alirocumab and evolocumab: a systematic review and meta-analysis of randomized controlled trials. Eur Heart J.

[64] Guedeney P, Sorrentino S, Giustino G (2021). Indirect comparison of the efficacy and safety of alirocumab and evolocumab: a systematic review and network meta-analysis. Eur Heart J Cardiovasc Pharmacother.

[65] Goldman A, Raschi E, Cukierman-Yaffe T (2022). Hyperglycaemic disorders associated with PCSK9 inhibitors: a real-world, pharmacovigilance study. Eur J Prev Cardiol.

[66] Ji C, Bai J, Zhou J, Zou Y, Yu M. Adverse event profiles of PCSK9 inhibitors alirocumab and evolocumab: data mining of the FDA adverse event reporting system. Br J Clin Pharmacol. 2022. 10.1111/bcp.15460. 10.1111/bcp.15460 35818959

[67] Olsson AG, Istad H, Luurila O (2002). Effects of rosuvastatin and atorvastatin compared over 52 weeks of treatment in patients with hypercholesterolemia. Am Heart J.

[68] Swanson SA, Tiemeier H, Ikram MA, Hernan MA (2017). Nature as a trialist?: deconstructing the analogy between Mendelian randomization and randomized trials. Epidemiology.

[69] Ference BA, Holmes MV, Smith GD: Using Mendelian randomization to improve the design of randomized trials. Cold Spring Harb Perspect Med 2021, 11(7). 10.1101/cshperspect.a040980 PMC8247560 33431510

[70] Ramin-Mangata S, Wargny M, Pichelin M (2020). Circulating PCSK9 levels are not associated with the conversion to type 2 diabetes. Atherosclerosis.

